# A method for increasing electroporation competence of Gram-negative clinical isolates by polymyxin B nonapeptide

**DOI:** 10.1038/s41598-022-15997-8

**Published:** 2022-07-08

**Authors:** Jilong Qin, Yaoqin Hong, Karthik Pullela, Renato Morona, Ian R. Henderson, Makrina Totsika

**Affiliations:** 1 Centre for Immunology and Infection Control, School of Biomedical Sciences, Queensland University of Technology, Brisbane, QLD Australia; 2 Institute for Molecular Bioscience, The University of Queensland, Brisbane, QLD Australia; 3 School of Biological Sciences, Department of Molecular and Biomedical Sciences, Research Centre for Infectious Diseases, University of Adelaide, Adelaide, Australia

**Keywords:** Microbiology, Microbial genetics, Biological techniques, Gene delivery, Genetic techniques, Microbiology techniques

## Abstract

The study of clinically relevant bacterial pathogens relies on molecular and genetic approaches. However, the generally low transformation frequency among natural isolates poses technical hurdles to widely applying common methods in molecular biology, including transformation of large constructs, chromosomal genetic manipulation, and dense mutant library construction. Here we demonstrate that culturing clinical isolates in the presence of polymyxin B nonapeptide (PMBN) improves their transformation frequency via electroporation by up to 100-fold in a dose-dependent and reversible manner. The effect was observed for PMBN-binding uropathogenic *Escherichia coli* (UPEC) and *Salmonella enterica* strains but not naturally polymyxin resistant *Proteus mirabilis*. Using our PMBN electroporation method we show efficient delivery of large plasmid constructs into UPEC, which otherwise failed using a conventional electroporation protocol. Moreover, we show a fivefold increase in the yield of engineered mutant colonies obtained in *S. enterica* with the widely used lambda-Red recombineering method, when cells are cultured in the presence of PMBN. Lastly, we demonstrate that PMBN treatment can enhance the delivery of DNA-transposase complexes into UPEC and increase transposon mutant yield by eightfold when constructing Transposon Insertion Sequencing (TIS) libraries. Therefore, PMBN can be used as a powerful electropermeabilisation adjuvant to aid the delivery of DNA and DNA–protein complexes into clinically important bacteria.

## Introduction

Delivery of foreign DNA into Gram-negative bacteria is a routine procedure in molecular microbiology and is essential for gene cloning, mutagenesis, and construction of mutant libraries. The most commonly used method for DNA delivery into bacterial cells is chemical transformation which involves cell washes with Mg^2+^ and/or Ca^2+^ under ice-cold conditions followed by heat-shock treatment^1–3^. Despite the high efficiency of chemical transformation for delivering DNA into laboratory adapted Gram-negative bacteria, especially *Escherichia coli* K-12 strains, it has limited success with natural and pathogenic isolates^4^. For this reason, almost all DNA delivery approaches for pathogenic bacteria solely rely on electrotransformation which involves washing bacterial cells with 10% (v/v) glycerol water followed by electro-permeabilisation at high voltages^5,6^. However, electrotransformation efficiency can remain low due to the presence of surface polysaccharides in some pathogenic bacteria, which act as a major barrier in successfully employing current DNA delivery methods^7^. In particular, high-efficiency transformation of Gram-negative pathogenic bacteria with either large DNA constructs (> 10 kb) or with DNA–protein complexes (e.g. for transposon mutant library generation) remains a challenge that limits their wider application across strains and species.

The exact molecular mechanism of DNA passage through bacterial membranes is still incompletely understood, yet it has been suggested that both calcium heat-pulse induced transformation and electroporation likely involve DNA transport by disrupting membrane lipids which may form channels^8,9^. Yet the Polymyxin B nonapeptide (PMBN) which is a membrane lipid disturbing agent was previously reported to have no effect on *E. coli* K-12 transformation by the calcium heat-pulse method, except at high DNA concentrations (~ 1 mg/ml) where it induced a small increase in transformation frequency^10^. PMBN is a cationic cyclic peptide that is devoid of the fatty acid tail of polymyxin B and is not bactericidal^11^. PMBN was shown to increase the permeability of the outer membrane (OM) of certain Gram-negative bacteria (e.g. *Salmonella enterica*, and *E. coli*) towards hydrophobic antibiotics^12^, presumably via direct binding to lipid A components in the bacterial OM and disruption of the asymmetric lipid bilayer^13,14^. Despite these properties being known for over 30 years, PMBN has never been tested for its ability to enhance bacterial transformation by electroporation, the primary method used for efficient delivery of DNA into pathogenic bacteria. Here, we present an optimised protocol using PMBN for improved electrocompetence of pathogenic Gram-negative bacteria, including multidrug-resistant strains. Our method is unique in its use of PMBN to reversibly increase large plasmid delivery, mutagenesis by allelic exchange, and insertional mutant library construction in clinically relevant bacteria and could form the basis for the wider application of these methodologies across bacteria.

## Results

### Growth of UPEC in the presence of PMBN increases electrotransformation efficiency

**Figure 1:**
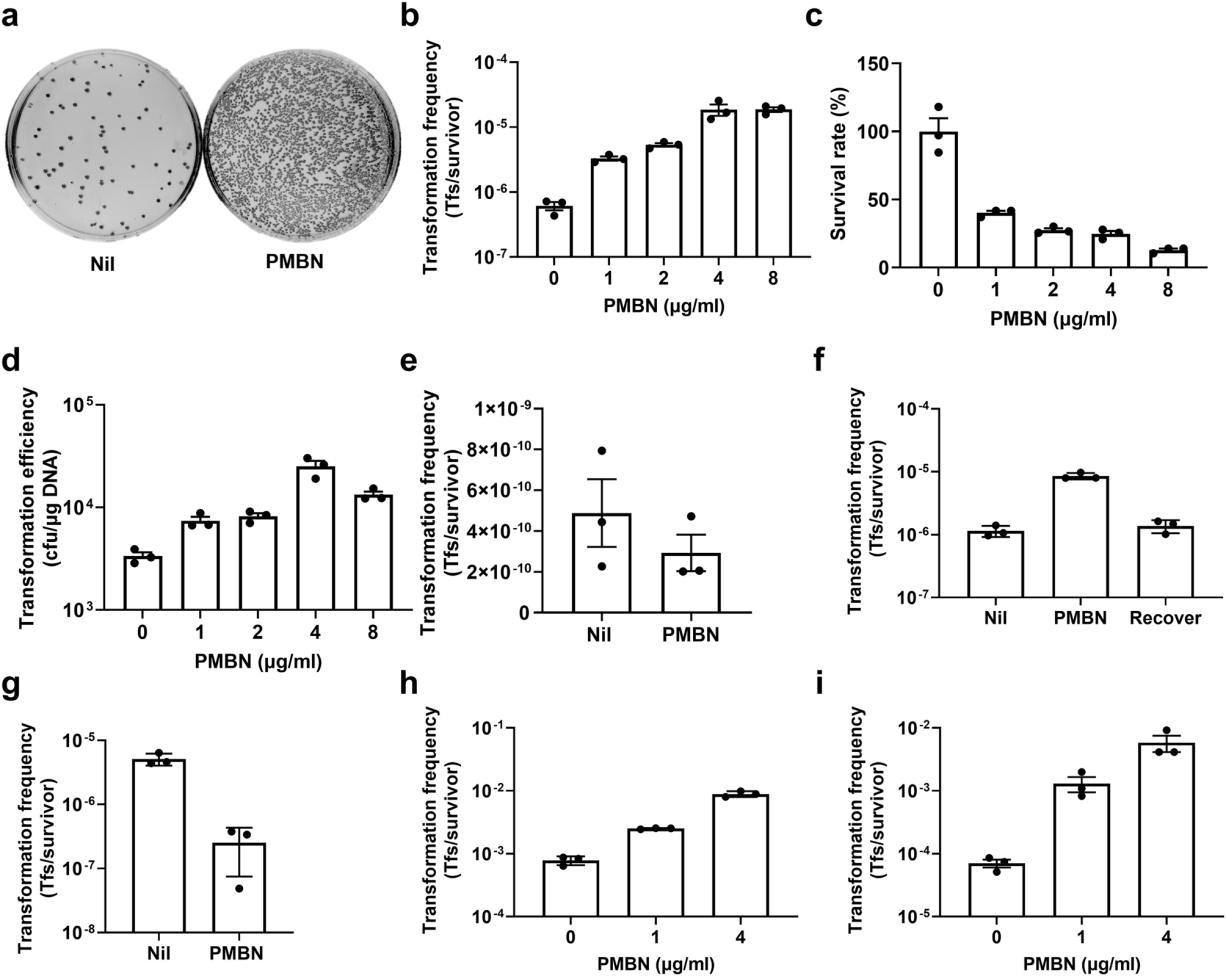
UPEC growth in the presence of PMBN increases cell electrocompetence in a reversible and dose dependent manner. (**a**) Representative plate images comparing the yield of pSU2718 plasmid electrotransformants of UPEC strain CFT073 grown without (Nil) or with PMBN (6 µg/ml) prior to electroporation. (**b**) Transformation frequency, (**c**) survival rate after electroporation, and (**d**) transformation efficiency of CFT073 grown with different concentrations of PMBN. (**e**) Chemical transformation frequency of CFT073 grown in the absence (Nil) or presence of PMBN (6 µg/ml). (**f**) Electrotransformation frequency of CFT073 grown in the presence of PMBN (4 µg/ml) and then sub-cultured into media devoid of PMBN prior to electroporation (Recover). (**g**) Electrotransformation frequency of CFT073 grown to OD_600_ ~ 0.7 and subsequently incubated without (Nil) or with PMBN (1 µg/ml) for 10 min before harvesting and electroporation. Electrotransformation frequency of UPEC strains UTI89 (**h**) and EC958 (**i**) grown without or with different concentrations of PMBN. In all experiments, the starting competent cell density was the same between groups. Data from three independent experiments are shown with bars denoting the group mean and error bars the standard error of the mean (mean ± SEM). *Tfs* transformants.

To test whether PMBN could increase the electrotransformation frequency of non-K-12 pathogenic *E. coli* strains, we initially grew reference uropathogenic *E. coli* (UPEC) strain CFT073 in the presence or absence of PMBN and performed electroporation with standard cloning vector pSU2718. CFT073 is a reference pyelonephritis strain that despite being genetically malleable^15,16^ was previously reported to be relatively low-transformable^4^. Culturing CFT073 in the presence of PMBN drastically increased the number of recovered transformants ~ 50-fold (Fig. 1a) with no noticeable impact on growth rate (Figure S1). An increase in electrotransformation frequency was observed by PMBN that was dose-dependent and highest after treatment of CFT073 with ≥ 4 μg/ml PMBN (Fig. 1b). Despite no impact on growth rate, we noticed PMBN treatment negatively affected cell survival after electroporation in a concentration-dependent manner, with less than 30% of cells treated with 4 μg/ml PMBN surviving post-electroporation (Fig. 1c). To confirm the observed PMBN effects on CFT073 electrocompetence, we also determined the transformation efficiency of CFT073 at different PMBN concentrations, which directly reflects the yield of transformants per DNA added. Despite the PMBN decrease in post-electroporation cell survival, transformation efficiency was still higher by ~ 10 fold for CFT073 cells treated with PMBN at 4 µg/ml over untreated controls (Fig. 1d), strongly indicating that cells have enhanced electrocompetence under these conditions. Culturing CFT073 in the presence of PMBN did not affect its chemical transformation frequency by the calcium heat-shock transformation method (Fig. 1e), consistent with a previous report using other *E. coli* strains^10^.

In order to examine if the observed PMBN effects on CFT073 cells were permanent, we tested the electrotransformation frequency of bacterial cells grown in the presence of PMBN and then sub-cultured in the absence of PMBN prior to electroporation. In the absence of maintained growth in PMBN containing media, the electrotransformation frequency of CFT073 reverted to the lower frequency of the untreated control (Fig. 1f). Brief exposure of CFT073 to PMBN was not sufficient to enhance electrocompetence, with addition of PMBN for only 10 min of culture prior to harvesting the bacterial cells in fact reducing the electrotransformation frequency compared to untreated controls (Fig. 1g). This suggests that bacterial growth in the presence of PMBN is required to enhance electrocompetence, potentially implying that rearrangement of cellular components may be induced in response to PMBN.

Similar increases in electrotransformation frequency were observed for two other UPEC strains belonging to different *E. coli* lineages: reference cystitis isolate UTI89 and reference multi-drug resistant isolate EC958 (Fig. 1h,i, respectively). For all tested strains the PMBN-induced effects were dose dependent, with EC958 showing the highest increase (~ 100-fold) in electrotransformation frequency over untreated controls. Taken together, our findings indicate that there is a threshold of electrocompetence induced by PMBN for increased plasmid delivery and that an optimal PMBN concentration can be determined for different host strains to achieve the highest transformant yield.

### PMBN increases electrotransformation frequency of other Gram-negative bacteria

**Figure 2:**
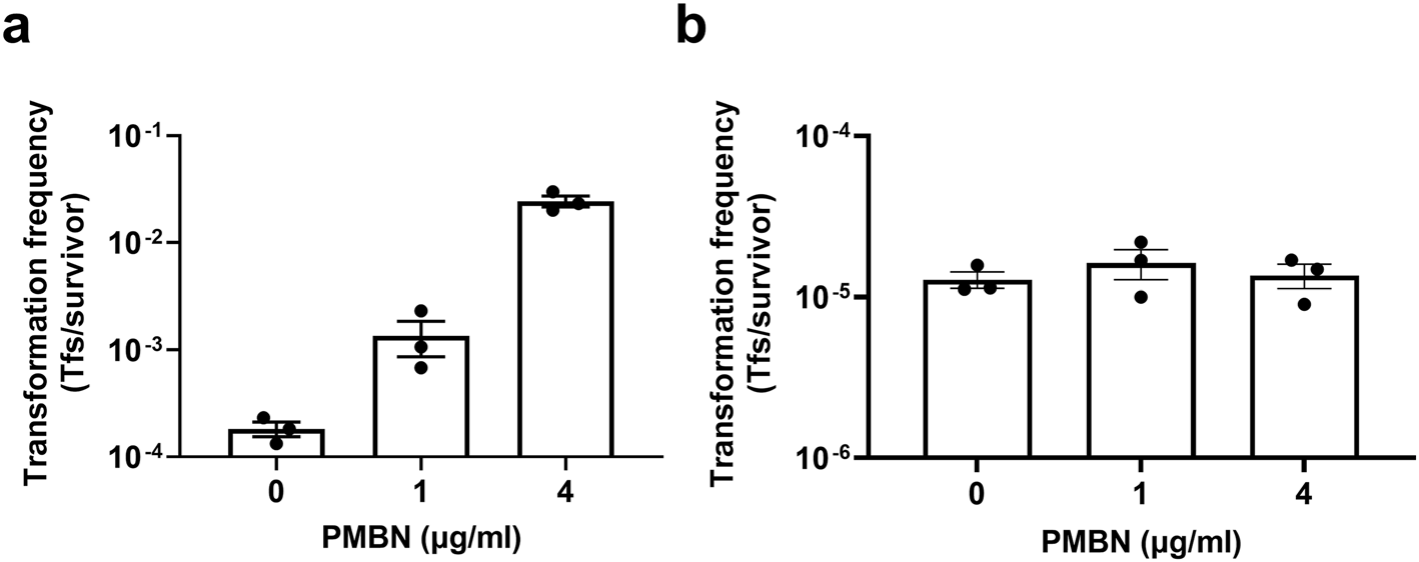
PMBN-mediated effects on electrotransformation frequency of *S.* *typhimurium* and *P. mirabilis*. Electrotransformation frequency of *S.* Typhimurium SL1344 (**a**), and uropathogenic *P. mirabilis* strain PM54 (**b**) grown in different concentrations of PMBN. In all experiments, the starting competent cell density was the same between groups. Data from three independent experiments are shown with bars denoting the group mean and error bars the standard error of the mean (mean ± SEM). *Tfs* transformants.

To test the effects of PMBN on the electrocompetence of other clinically relevant pathogens, we selected *Salmonella enterica* serovar Typhimurium strain SL1344 and *Proteus mirabilis* clinical isolate PM54 to culture in the presence of PMBN for electrotransformation with vector pSU2718. A dose dependent increase in SL1344 transformation frequency was observed with PMBN, with ~ 100-fold increase in electrotransformation frequency obtained for PMBN treated cells at 4 µg/ml over untreated controls (Fig. 2a). In contrast, the electrocompetence of *P. mirabilis* PM54 was unaffected by PMBN at all concentrations tested (Fig. 2b). This finding is in agreement with a previous study reporting that binding of PMBN to *P. mirabilis* is inefficient. It is suggested that this is due to a less acidic LPS that confers on this species resistance to polycationic peptides^13^.

### PMBN improves delivery of large plasmids into clinical isolates

**Figure 3:**
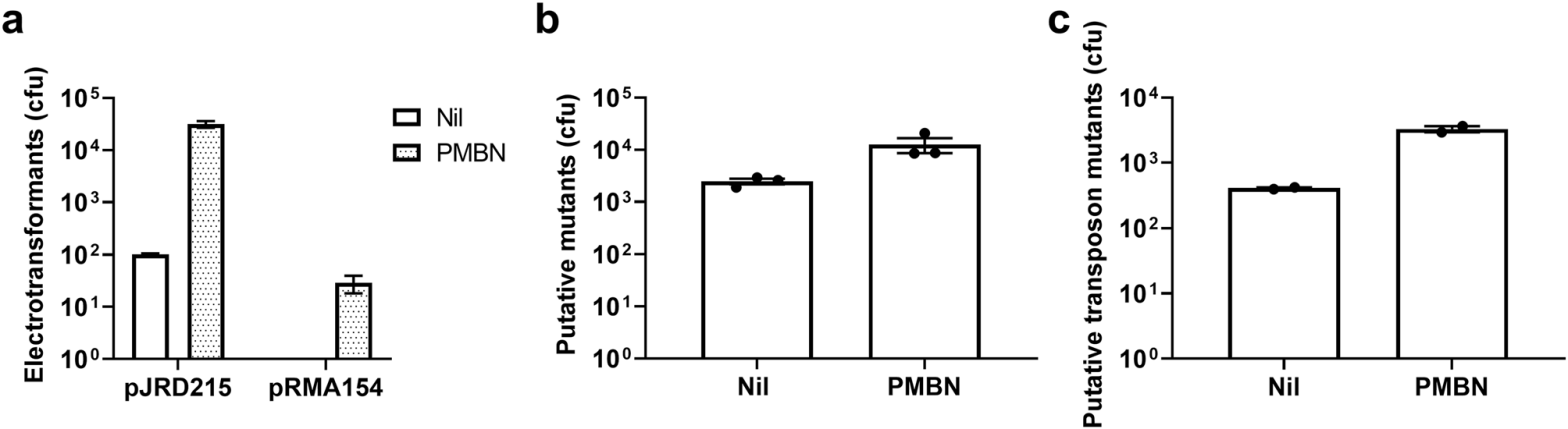
Application of the PMBN electroporation method to improving delivery of large size DNA, mutagenesis rates and TIS library yields in Gram-negative clinical isolates. (**a**) Number of UPEC CFT073 electrotransformants with large plasmid constructs recovered from cultures prepared without (Nil) or with PMBN (1 μg/ml). (**b**) Comparison of putative *ΔoafA::cat* mutant cfu yields following λ-Red mutagenesis of *S.* Typhimurium SL1344 electrocompetent cells without (Nil) or with 1 μg/ml PMBN. (**c**) Comparison of putative transposon mutant cfu yields in UPEC UTI89 following standard electroporation without PMBN (Nil) or with PMBN (3 μg/ml) treatment. In all experiments, the starting competent cell density was the same between groups. Data from three (**a**,**b**) or two (**c**) independent experiments are summarised in graphs showing mean ± SEM.

Investigation of virulence factors in pathogenic Gram-negative bacteria often requires construction of large plasmids containing entire gene operons that typically exceed 10 kb^17^, such as fimbrial and O antigen gene clusters^18,19^. Due mainly to their low transformation efficiency, these large constructs are typically studied in the highly transformable *E. coli* K-12 background rather than the relevant (but also more technically challenging) background of the isolates that encode them. Therefore, we examined if PMBN-grown electrocompetent cells of UPEC strain CFT073 showed improved uptake of two previously constructed large constructs: pJRD215^20^ (10.2 kb) and pRMA154 (22.5 kb)^19^. The number of pJRD215 containing electrotransformants was more than 100-fold higher from PMBN treated cells over untreated controls (Fig. 3a). For the larger pRMA154 construct, a small number of transformants was only obtained with PMBN-grown electrocompetent cells, while no colonies were recovered from CFT073 cells grown in the absence of PMBN (Fig. 3a). These data demonstrate that PMBN can be employed to overcome the challenge of delivering large constructs into pathogenic strains.

### PMBN increases the yield of bacterial mutants obtained with the widely used lambda-Red recombineering system

Allelic exchange mediated by λ-Red proteins remains as a common method to make chromosomal modifications in Gram-negative bacteria^21^. Typically, this method uses a host strain carrying a plasmid encoding the λ-Red recombination genes under an arabinose inducible promoter and electroporation of this host strain with a dsDNA PCR product carrying selection marker flanked by short homologous regions of target genes. Despite its success in recombineering DNA in laboratory *E. coli* strains, the success rate of genetic engineering in pathogenic strains is normally > 100-fold lower due to their low transformability^4^ and strong efflux of arabinose via drug-efflux pumps expressed especially in multi-drug resistant pathogenic *Salmonella* strains^22^. Therefore, we tested if PMBN could improve the mutant yield of λ-Red recombination by mutating the *oafA* gene in reference *S.* Typhimurium strain SL1344 as an example. Using an optimised λ-Red mutagenesis protocol, we obtained a fivefold increase in the yield of putative *ΔoafA::cat* mutant colonies when electro-competent cells were prepared by culture in media supplemented with 1 μg/ml PMBN (Fig. 3b). PCR screening of 30 putative mutant colonies confirmed correct *oafA* deletion with 100% success rate in both groups (data not shown).

### PMBN increases the efficiency of constructing dense transposon mutant libraries

Advances in next-generation genome sequencing technologies have allowed the development of Transposon Insertion Sequencing (TIS) as a powerful tool to screen for bacterial genes that are essential for cell survival and fitness under different conditions^23^. TIS library construction may involve electroporation of a protein-DNA complex, i.e. transposome, into bacterial cells that contains the transposase enzyme and transposon DNA encoding antibiotic resistance markers^24^. In order to generate a master library with adequate insertion density across the entire bacterial genome^23^, TIS-based methods require the pooling of multiple mutagenesis sub-libraries especially in pathogenic strains with low transformation frequency. As a result, the generation of dense transposon mutant libraries (ca. 100,000 mutants/Mb) across diverse bacteria and clinical isolates is often the limiting step in the wider application of TIS-based methodologies. Therefore, we tested if PMBN could enhance the delivery of protein-DNA complexes into a clinical UPEC strain to facilitate more efficient construction of dense TIS libraries. Intriguingly, we achieved an eightfold increase in the insertional mutant yield of each electrotransformation reaction when UPEC strain UTI89 was grown in the presence of PMBN (3 μg/ml) (Fig. 3c). These data suggest that PMBN can improve the delivery of DNA–protein complexes into pathogenic Gram-negative bacteria.

## Discussion

In this study we present an electrotransformation protocol optimised to enhance the electrocompetence of clinically relevant Gram-negative bacteria using the cationic cyclic peptide PMBN. We show that PMBN enhanced the electrotransformation frequency of several Gram-negative bacteria in a dose dependent manner, achieving improvement of up to 100-fold. We also demonstrate how this protocol is readily applicable to enhance commonly used molecular biology methods involving DNA and DNA–protein complex delivery into bacterial cells.

The study of large genetic elements in bacteria (e.g. operons, genomic islands and virulence or antibiotic resistance plasmids) requires the successful delivery of large plasmid constructs in a clinically relevant host background and this still remains a challenge in the field as demonstrated here. Conventionally triparental bacterial conjugation^25^ could be used, but this requires re-engineering of the host vector and is restricted to recipient strains that lack conjugation exclusion factors^26,27^. Here we have demonstrated successful delivery of a large plasmid construct (25 kb) encoding a gene cluster responsible for the synthesis of a heterologous O antigen in a pathogenic *E. coli* background. We have also demonstrated a fivefold increase in the yield of chromosomal mutants in a widely studied pathogenic *S*. Typhimurium strain. The improvement in the mutagenesis rate we observed is likely attributed to two factors: (a) enhanced DNA delivery into the cells, as demonstrated by our study findings and (b) PMBN could also function as a membrane permeabilising agent to counteract TolC mediated efflux of l-arabinose, thereby allowing more DNA template delivery as well as higher-level of induced expression of the lambda-Red proteins, resulting in an increased allelic exchange rate. The latter is supported by a previous reported finding that a *tolC* knockout mutant had reduced efflux of l-arabinose^22^.

Our improved mutagenesis protocol using PMBN could also enable construction of chromosomal gene mutant libraries. This could be done by: (1) acquiring a target gene knockout with a positive–negative selection system^28^ by the standard lambda-Red mutagenesis method; (2) generating target gene templates with random mutations via error-prone PCR; (3) performing a second allelic exchange reaction using our PMBN enhanced mutagenesis protocol to deliver target gene DNA amplicons with random mutations; and (4) selecting putative flawless allelic mutants on an appropriate negative selection media. An improved yield of mutants by employing our PMBN method will enable efficient construction of a mutant gene library at the chromosomal level so that the study of the mutant alleles is not subject to plasmid copy number or expression differences. Lastly, we have shown that PMBN enhanced electrotransformation can be used to optimise the delivery of DNA–protein complexes (e.g. for TIS library construction) and we expect that this could also potentially be extended to developing a delivery system for ribonucleoprotein (RNP)-mediated CRISPR genome editing^29^ in bacterial cells for efficient targeted mutagenesis.

Interestingly, our findings with PMBN and pathogenic *E. coli* and *S.* Typhimurium strains, did not extend to a clinical *P. mirabilis* strain. *P. mirabilis* is naturally resistant to PMBN-induced antibiotic sensitising, in contrast to various *E. coli* and *S.* Typhimurium strains that were shown to be sensitised towards a set of antibiotics following treatment with PMBN^12^. PMBN was shown to readily bind to *E. coli* and *Salmonella* membranes at a level of 3–5 μg/mg dry weight bacteria, whereas *P. mirabilis* membranes do not bind PMBN^13^. This would imply that the enhanced electrotransformation competence we observed requires PMBN to bind to the membrane and we thus predict that this protocol would have limitations with other naturally polymyxin-resistant bacteria such as *Proteus vulgaris, Morganella morganii, Providencia stuartii* and *Serratia marcescens,* but could be used to enhance electroporation competence of polymyxin susceptible bacteria, such as *Klebsiella pneumoniae, Klebsiella oxytoca, Enterobacter cloacae, Enterobacter agglomerans Acinetobacter calcoaceticus, Pseudomonas aeruginosa* and *Pseudomonas maltophilia*^12^.

We also observed that growing pathogenic *E. coli* in the presence of PMBN made the electrocompetent cells more vulnerable to high voltage. We speculate that this is likely due to PMBN enhancing membrane damage induced by electroporation (thought to transitionally cause formation of aqueous pores on membranes and even membrane rupture^30^) by binding to lipid A and disrupting the asymmetric bilayer of the bacterial OM. In future, the survival rate of PMBN-grown cells could be improved by exploring conditions that promote efficient repair of cell membrane damage in the cell. It is important to note however that the enhanced electrotransformation cell competence by PMBN is likely not solely due to its membrane disrupting effects, as short exposure of bacterial cells to PMBN at early/mid-exponential phase instead reduced electrotransformation frequency. This, combined with the fact that PMBN treatment had no effect on plasmid chemical transformation frequency shown here and previously^10^, while it increased the electrotransformation frequency (and efficiency) of treated mid-exponential phase cells, suggests that bacterial growth in the presence of PMBN may be required to induce membrane modifications. Indeed, PMBN was previously reported to be sensed by the bacterial two component PhoPQ system^31^ and induce PmrAB-mediated gene expression^32^ to modify lipid A in the OM. Such modifications might be related to the enhanced electrotransformation frequencies we observed and a mechanistic investigation into PMBN-induced OM modifications constitutes a future investigation. Irrespective of the exact nature of the PMBN induced membrane changes, they seem to be transient and reversible as cells no longer exposed to PMBN revert to lower electrotransformation frequencies similar to PMBN untreated cells.

To our knowledge, this is the first study to show enhanced DNA delivery into clinically relevant Gram-negative bacteria using a cationic membrane binding peptide. The method presented here can be readily adapted for improving other genetic manipulation techniques involving the delivery of exogenous DNA into natural isolates. Therefore, we propose PMBN as a powerful Gram-negative membrane electropermeabilisation adjuvant for achieving enhanced DNA delivery into bacterial cells by electroporation.

## Materials and methods

### Bacterial strains and plasmids

The bacterial strains and plasmids used in this work are listed in Table S1. Single colonies of bacterial strains grown overnight on Lennox Broth (LB)^33^ agar (1.5% w/v) plates were picked and grown overnight in LB at 37 °C for subsequent experiments. For *Proteus mirabilis*, modified plates with LB 3% (w/v) agar and no NaCl were used to prevent swarming. Where appropriate, media were supplemented with ampicillin (Amp, 100 µg/ml), kanamycin (Kan, 50 µg/ml) or chloramphenicol (Chl, 25 µg/ml).

### Preparation of electrocompetent cells and electroporation of plasmid DNA

Bacterial cells grown overnight in 10 ml LB at 37 °C were sub-cultured 1 in 100 into 10 ml LB supplemented with 1, 2, 3, 4, 6, or 8 μg/ml of PMBN (prepared in 5 mg/ml in Milli Q water, Sigma, P2076) to mid-exponential phase (until all cultures reached an identical optical density at 600 nm (OD_600_) between 0.6 and 0.8). Bacterial cells were harvested via centrifugation (5000×*g*, 5 min, 4 °C), washed twice with 5 ml of ice-cold 10% (v/v) glycerol water and resuspended in 200 μl ice-cold 10% (v/v) glycerol water. Unless otherwise stated, 100 ng of pSU2718 was used as a standard for all electrotransformation (quantity of all other plasmids used in this study was 100 ng per electrotransformation; Table S1) and mixed with 100 μl of competent cells and electroporated at 2.5 kV in an electroporation cuvette with 2 mm gap. To determine the transient effect of PMBN on electrotransformation, mid-exponential cells grown in the presence of PMBN were then sub-cultured 1 in 100 into 10 ml LB without PMBN and grown to mid-exponential phase for electroporation as described above. Electroporated cells were immediately recovered with 900 µl of LB and incubated at 37 °C with shaking (200 rpm) for 45 min before spreading onto LB agar plates containing appropriate antibiotics (to determine number of transformants) or nonselective media (to determine number of survivor cells post-electroporation). Recovery plates were incubated at 37 °C for 16 h. To determine cell survival rates, cultures were sampled immediately before and after electroporation and serial dilutions were plated on LB agar plates incubated overnight at 37 °C for enumeration of viable colony forming units (CFUs). Electrotransformation frequency was estimated as the number of transformants obtained per the number of viable cells after electroporation (tfs/survivor)^6^. Transformation efficiency was defined as the number of transformants per μg of DNA (cfu/μg DNA).

### Preparation of chemically competent cells and chemical transformation of plasmid DNA

Bacterial cells grown in 10 ml LB at 37 °C were sub-cultured 1 in 100 into 10 ml LB with or without 6 μg/ml of PMBN and cultured to an identical OD_600_ value in mid-exponential phase (OD_600_ ~ 0.6 to 0.8). Cells were harvested via centrifugation (5000×*g*, 5 min), sequentially washed and incubated on ice with 10 ml of 100 mM MgCl_2_ for 1 h, 10 ml of 100 mM CaCl_2_ for 1 h, and finally resuspended into 0.3 ml of 85 mM CaCl_2_, 15% (v/v) glycerol. For chemical transformation, 100 μl of chemically competent cells were mixed with 100 ng of pSU2718 plasmid DNA and incubated on ice for 20 min, heat-shocked at 42 °C for 30 s and rested on ice for 5 min. 900 μl of LB was then added and transformed cells were incubated at 37 °C with aeration (200 rpm) for 45 min before spreading (100 μl) onto LB agar plates containing appropriate antibiotics. Transformation frequency was determined as above.

### Bacterial mutagenesis via allelic exchange

Mutagenesis was performed as described previously^21,34^ with laboratory adapted optimisations. Briefly *Salmonella enterica* serovar Typhimurium strain SL1344 harbouring plasmid pKD46 grown overnight in 10 ml LB at 30 °C was sub-cultured into 25 ml LB in a 250 ml flask supplemented with or without 1 μg/ml of PMBN. Expression of the lambda phage-derived Red proteins were then induced with 50 mM l-arabinose at OD_600_ of 0.3. Bacterial cells were then harvested at OD_600_ of 0.6–0.8 by directly mixing 15 ml cultures with 20 ml of 10% (v/v) ice-cold glycerol water followed by centrifugation. Bacterial cells equivalent to OD_600_ of 2 in 1 ml were then taken and washed twice with 10% (v/v) ice-cold glycerol and finally resuspended in 100 μl of 10% (v/v) ice-cold glycerol for subsequent electroporation. The *cat* gene was PCR amplified from pKD3 using Taq DNA Polymerase with ThermoPol Buffer (New England BioLabs, M0267) following the manufacturer’s protocol and primers containing 50 bp up- and down-stream sequences homologous to the *oaf* target gene (Table S1). The amplicon was then treated with DpnI (New England BioLabs, R0176) at 37 °C for 1 h, purified using QIAquick PCR Purification Kit (Qiagen, 28104) and quantified by NanoPhotometer N60/N50 (Implen).

Purified PCR amplicon (1.5 μg) was then introduced into electrocompetent cells via electroporation as above and cells were immediately recovered in 3 ml LB in a 50 ml Falcon tube that was incubated for 2 h at 37 °C before plating out (100 μl) on LB agar plates supplemented with 17 μg/ml chloramphenicol. Plates were incubated at 37 °C for 16 h to acquire chloramphenicol resistant colonies. Samples (10 μl) immediately before and after electroporation were serially diluted and spot plated onto LB agar plates, incubated at 37 °C overnight to determine viable cells. Candidate mutant colonies were picked and patched onto LB agar supplemented with chloramphenicol, and cell lysates were prepared in 100 μl water by heating to 100 °C for 5 min. Lysate DNA was screened by PCR using primers annealing to regions flanking the amplicon insertion site (Table S1).

### Transposon insertion sequencing (TIS) library construction

TIS library construction was done as previously described^23^. Briefly, a single colony of UPEC strain UTI89 was inoculated in 5 ml of LB broth and grown overnight at 37 °C shaking at 180 rpm. The overnight was used to inoculate into 15 ml of 2 × TY medium (1:100) and grown at 37 °C in the absence or presence of 3 μg/ml PMBN shaking at 180 rpm until the culture reached an OD_600_ between 0.65 and 0.78. Culture volume equivalent to OD_600_ of 5 in 1 ml was collected and the cells were then harvested by centrifugation at 2410×*g*. The cell pellet was washed with twice equal culture volume of 10% (v/v) ice cold glycerol twice at 2410×*g* at 4 °C, and then resuspended in 100 µl of 10% (v/v) glycerol to prepare electrocompetent cells. The UTI89 electrocompetent cells were then incubated briefly with 0.2 μl of Kan2-Tn5-mini transposome (Lucigen, TSM99K2) and electroporated using a 2 mm gapped cuvette using 2.5 kV of voltage. After electroporation, the cells were recovered by adding 900 µl of 37 °C pre-warmed SOC medium^1^ and incubating for 2 h at 37 °C with shaking at 180 rpm. Serial dilutions were prepared from the electroporated samples before and after recovery and plated on LB plates with no antibiotics and on 35 μg/ml Kanamycin containing LB plates, respectively.

## Supplementary Information

Supplementary Information.

## Data Availability

All data generated or analysed during this study are included in this published article (and its Supplementary Information files).
