# Supplementary material for: A method for increasing electroporation competence of Gram-negative clinical isolates by polymyxin B nonapeptide
Source: Sci Rep. 2022 Jul 8;12:11629. doi: 10.1038/s41598-022-15997-8 (PMC9270391; doi:10.1038/s41598-022-15997-8)
Supplement: Supplementary file 1 — Supplementary Information. [file 41598_2022_15997_MOESM1_ESM.pdf]

Supporting Information

A method for increasing electroporation competence of Gram-negative clinical isolates by Polymyxin B nonapeptide

Jilong Qin[1]*, Yaoqin Hong[1], Karthik Pullela[2], Renato Morona[3], Ian R. Henderson[2], Makrina Totsika[1]*.

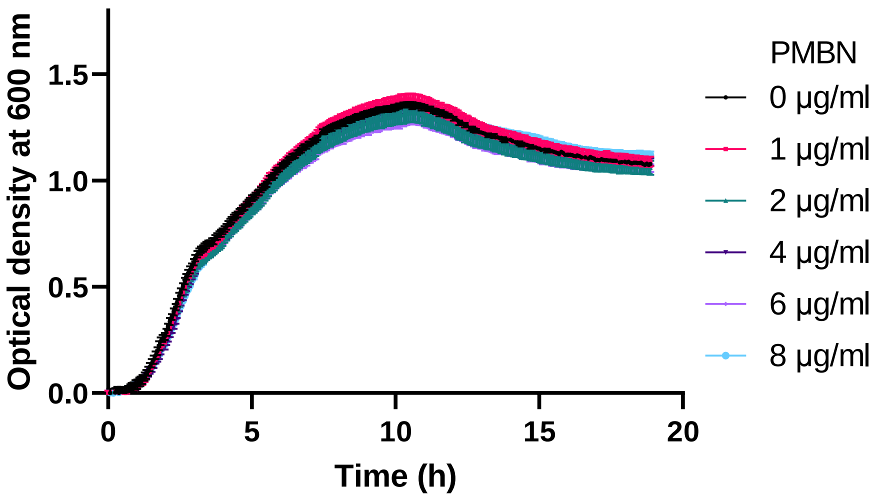

Figure S1. Growth curves of UPEC strain CFT073 in the absence or presence of PMBN at different concentrations. Overnight cultures of CFT073 were diluted 1 in 100 in 700 µl of LB media in a 24-well plate supplemented without or with PMBN at different concentrations as indicated. Plates were incubated at 37 °C in a CLARIOstar plate reader (BMG, Australia) programmed to measure the absorbance (O.D. 600 nm) every 6 minutes over 18 h. Data points represent the mean OD_{600} from three independent experimental repeats with error bars showing the standard error of the mean.

Table S1. Strains, plasmids and oligonucleotides

Strains	Description	Source
MT542	Uropathogenic *Escherichia coli* isolate CFT073	[1]
MT4	Uropathogenic *E. coli* isolate UTI89	[2]
MT7	Uropathogenic *E. coli* isolate EC958	[3]
MT234	*Salmonella typhimurium* SL1344	[4]
MT1482	*Proteus mirabilis* clinical isolate PM54	[5]
Plasmids		
pSU2718	Cloning vector	[6]
pKD46	Temperature sensitive construct expressing λ-Red proteins	[7]
pKD3	Plasmid encoding FRT-flanked *cat* cassette	[7]
pJRD215	Wide-host-range cloning vector	[8]
pRMA154	pJRD215 encoding *S. flexneri* O antigen gene cluster	[9]
Primers		
534	SL1344 OafA KO Forward<br>CATTATCTTAATTTCGTCTTGTGTGGCACCTTGGAATTATAG<br>GTAAAAAGTGTAGGCTGGAGCTGCTTC	This work
535	SL1344 OafA KO Reverse<br>GCATTATTGTTGTAGTTTTATAAAATAAAAAGAGGGGCAA<br>GCCCCTCTGTATGGGAATTAGCCATGGTCC	This work
536	SL1344 OafA Check Forward<br>GAATGTGTCCGGTACCGCTT	This work
537	SL1344 OafA Check Reverse<br>GCCAACGAGCACATGAACAG	This work

1 Mobley, H. L. *et al.* Pyelonephritogenic Escherichia coli and killing of cultured human renal proximal tubular epithelial cells: role of hemolysin in some strains. *Infect Immun* **58**, 1281-1289, doi:10.1128/iai.58.5.1281-1289.1990 (1990).

2 Mulvey, M. A., Schilling, J. D. & Hultgren, S. J. Establishment of a persistent Escherichia coli reservoir during the acute phase of a bladder infection. *Infect Immun* **69**, 4572-4579, doi:10.1128/IAI.69.7.4572-4579.2001 (2001).

3 Totsika, M. *et al.* Insights into a multidrug resistant Escherichia coli pathogen of the globally disseminated ST131 lineage: genome analysis and virulence mechanisms. *PloS one* **6**, e26578, doi:10.1371/journal.pone.0026578 (2011).

4 Hoiseth, S. K. & Stocker, B. A. D. Aromatic-dependent Salmonella typhimurium are non-virulent and effective as live vaccines. *Nature* **291**, 238-239, doi:10.1038/291238a0 (1981).

5 Furlong, E. J. *et al.* A shape-shifting redox foldase contributes to Proteus mirabilis copper resistance. *Nat Commun* **8**, 16065, doi:10.1038/ncomms16065 (2017).

6 Martinez, E., Bartolome, B. & de la Cruz, F. pACYC184-derived cloning vectors containing the multiple cloning site and lacZ alpha reporter gene of pUC8/9 and pUC18/19 plasmids. *Gene* **68**, 159-162, doi:10.1016/0378-1119(88)90608-7 (1988).

7 Datsenko, K. A. & Wanner, B. L. One-step inactivation of chromosomal genes in Escherichia coli K-12 using PCR products. *Proceedings of the National Academy of Sciences* **97**, 6640-6645, doi:doi:10.1073/pnas.120163297 (2000).

8 Davison, J., Heusterspreute, M., Chevalier, N., Ha-Thi, V. & Brunel, F. Vectors with restriction site banks. V. pJRD215, a wide-host-range cosmid vector with multiple cloning sites. *Gene* **51**, 275-280, doi:10.1016/0378-1119(87)90316-7 (1987).

9 Morona, R., Mavris, M., Fallarino, A. & Manning, P. A. Characterization of the rfc region of Shigella flexneri. *J Bacteriol* **176**, 733-747, doi:10.1128/jb.176.3.733-747.1994 (1994).
